
JOURNAL INFORMATION
==============================
NLM Title Abbreviation: ScientificWorldJournal
Journal ID: ScientificWorldJournal
NLM Journal ID: 101131163
Journal ID: TSWJ

The Scientific World Journal

ISSN: 2356-6140
EISSN: 1537-744X
Publisher: Wiley

ARTICLE INFORMATION
==============================
PMCID: PMC5848701
PMID: 19082420
DOI: 10.1100/tsw.2008.155
Article ID: 106761
Article version: 1
Subjects: Directions in Science

Vascular Basement Membrane Components in Angiogenesis — An Act of Balance

Jakobsson Lars 1 *
Claesson-Welsh Lena 2 *
1 Vascular Biology Laboratory, London Research Institute, Cancer Research UK, 44 Lincolnʼs Inn Fields, London WC2A 3PX, UK
2 Uppsala University, Department of Genetics and Pathology, Rudbeck Laboratory, Dag Hammarskjöldsv. 20, 751 85 Uppsala, Sweden
*Lars Jakobsson: lena.claesson-welsh@genpat.uu.se*Lena Claesson-Welsh: lars.jakobsson@cancer.org.ukAcademic Editor: Martin Goette

Publication date: 2008
Electronic publication date: 2008 Dec 14
Volume: 8
First page: 1246
Last page: 1249
Received 2008 Aug 21; Revised 2008 Nov 27; Accepted 2008 Dec 2
Copyright: Copyright © 2008 Lars Jakobsson and Lena Claesson-Welsh.
Copyright year: 2008
License: This is an open access article distributed under the Creative Commons Attribution License, which permits unrestricted use, distribution, and reproduction in any medium, provided the original work is properly cited.
License URL: https://creativecommons.org/licenses/by/3.0/

Keywords: vasculogenesis, angiogenesis, basement membrane, laminin, lumen, flow, embryonic stem cells

Funding: Cancer Research UK Swedish Cancer Foundation Swedish Science Council

==============================
Directions in Science
*TheScientificWorldJOURNAL* (2008) 8, 1246–1249
ISSN 1537-744X; DOI 10.1100/tsw.2008.155

TheScientificWorldJOURNAL
www.thescientificworld.com

### Vascular Basement Membrane Components in Angiogenesis — An Act of Balance

Lars Jakobsson[1,]* and Lena Claesson-Welsh[2,]*

*[1]Vascular Biology Laboratory, London Research Institute, Cancer Research UK, 44 Lincoln's Inn Fields, London WC2A 3PX, U.K.; [2]Uppsala University, Department of Genetics and Pathology, Rudbeck Laboratory, Dag Hammarskjöldsv. 20, 751 85 Uppsala, Sweden*

**E-mail**: lena.claesson-welsh@genpat.uu.se; Lars.Jakobsson@cancer.org.uk

*Received August 21, 2008; Revised November 27, 2008; Accepted December 2, 2008; Published December 14, 2008*

---

**KEYWORDS:** vasculogenesis, angiogenesis, basement membrane, laminin, lumen, flow, embryonic stem cells

---

Angiogenesis is crucial in the progression of a number of pathological conditions, such as diabetic retinopathy, rheumatoid arthritis, psoriasis, and cancer[1]. In contrast to vessels in healthy tissues, the vasculature in these pathologies is highly unstable, constantly dissolving and renewing. Characteristically, vessels in pathologies have discontinuous basement membrane (BM) coverage[2]. The consequences of shifts in BM density and composition are still relatively unknown. As discussed below, several studies have illustrated that partial loss of the vascular BM during development results in the widening of vessels. This has been suggested to be a result of reduced mechanical resistance to the force inflicted by the blood pressure. However, recent data indicate that depletion of BM laminins (LMs) leads to enlarged vessels even in the absence of cardiac activity and blood pressure[3].

All endothelial cells (ECs) in the body rest on BMs composed of intricate networks of matrix proteins, such as fibronectins, collagen IV, and LMs. LMs are heterotrimeric glycoproteins composed of α-, β-, and γ-chains. The vascular BM has a unique composition with the presence of LM α-chains 4 and 5 that combine with the β1- and γ1-chains to form LM-411 and LM-511 (previously denoted laminin 8 and 10, respectively)[4,5].

The function of the vascular LMs has long been sought and their roles have mainly been addressed by gene inactivation (reviewed by Hallman et al.[6]). Deletion of the LM α5-chain (encoded by *lama5*) results in lethality between embryonic day 14 and 17.5, with failure of neural tube closure, severe brain and limb defects, and dysmorphogenesis of the placenta[7]. Interestingly, the great majority of the vasculature is largely unaffected in the *lama5-/-* embryos, but the placental vasculature is reduced, less branched, and shows an increase in vessel diameter. The *lama4-/-* (lacking the LM α4-chain) mice suffer from hemorrhages and display enlarged blood vessel width during embryonic and neonatal stages[8]. The vascular phenotype decreases after birth and is gradually lost. Up-regulation of the α5-chain or other LMs in the *lama4-/-* animals has not been detected. In a recent study[3], we examined the role of BM LMs in the formation of vascular structures in spheroids of differentiating embryonic stem cells, denoted embryoid bodies[9]. This experimental approach in many cases allows for the study of gene modifications that are incompatible with embryonic survival *in vivo*, or alternatively are clouded *in vivo* due to

*Corresponding authors.
©2008 with author.
Published by TheScientificWorld; www.thescientificworld.com functional compensation by related proteins. Deletion of the LMγ1-chain (*lamc1-/-*) in mice is incompatible with development beyond embryonic day 5.5, i.e., before onset of vascular development. However, in cultures of *lamc1*-/- embryoid bodies, vascular development proceeded. Analysis of the vascular BM revealed a total absence of extracellular LMs (13 out of 16 LM isoforms analyzed)[3], similar to what has been demonstrated for *lamc1-/-* epithelial BM[10]. Still, ECs formed and organized into three-dimensional vascular structures with apparently normal pericyte coverage. Interestingly, the prominent vascular phenotype in *lamc1-/-* embryoid bodies was widened vessel lumen (see Fig. 1 for a schematic illustration). Thus, widening of the vascular lumen is a common phenotype to deletion of the vascular BM LMs.

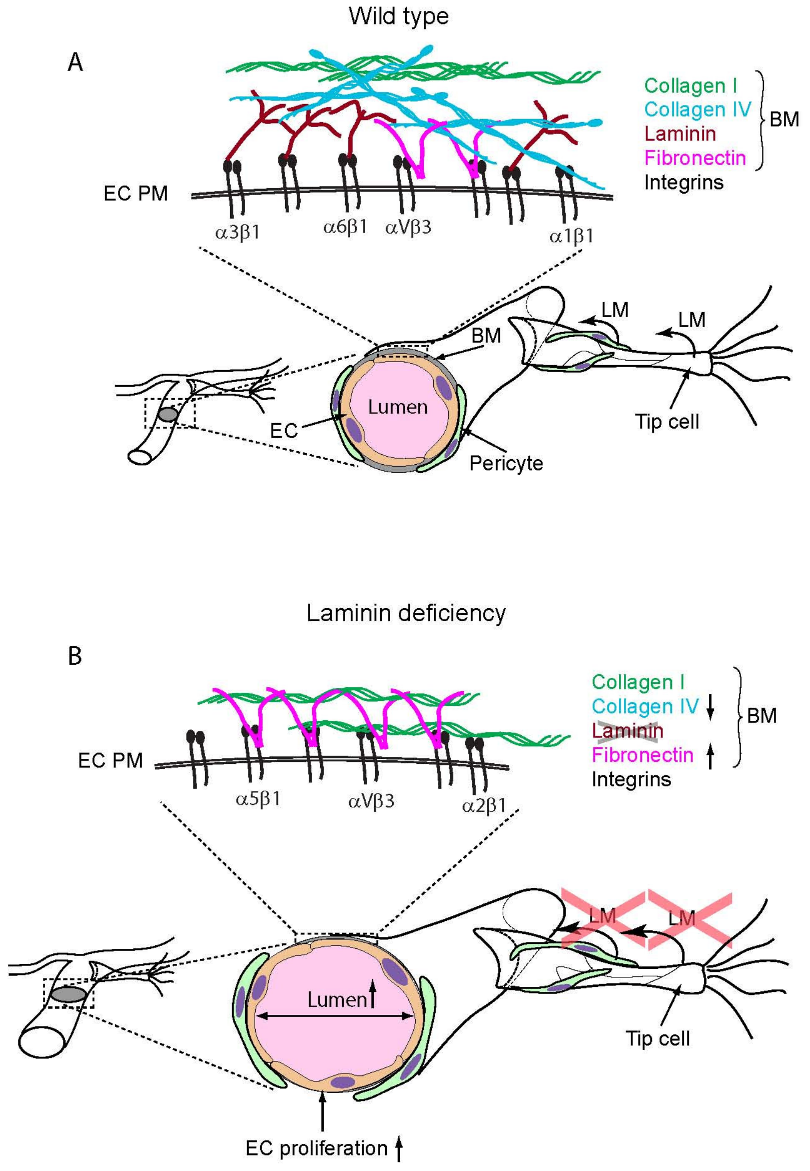

**FIGURE 1**. Schematic overview of sprouting vascular branches. (A) Wild-type vasculature. LMs secreted by pericytes as well as ECs in the tip and stalk initiate BM formation along the developing sprout. Top. Possible interactions between integrins, spanning the abluminal EC plasma membrane (PM), and certain molecules within the BM. (B) LMγ1-null vasculature. No LMs are deposited in the BM. Collagen IV is barely detectable. Increased fibronectin levels and increased EC proliferation contribute to a wider vessel lumen.

Since there is no flow in embryoid bodies, we conclude that widening of the vessel lumen in the absence of LM deposition is not a consequence of decreased resistance to shear stress or blood pressure in this model. What causes the widening of the vessel lumen in the absence of LMs? We could show that LM deficiency was accompanied by an increase in fibronectin and a decrease in collagen IV protein levels in the BM in the *lamc1-/-* embryoid bodies. It is possible that endothelial integrins become differentially activated as a consequence of this change in BM composition. Fibronectin is known to affect cellular morphology via its binding to β1 integrins. In accordance, *lamc1-/-* ECs were more spread out than wild-type ECs and, strikingly, the *lamc1-/-* phenotype was partially rescued by addition of purified LM protein. Also, *lamc1-/-* ECs sprouting into a surrounding collagen I matrix showed a higher rate of proliferation, which may reflect different integrin engagement. Since wide and thin vessels normally have roughly the same EC density (number of ECs/circumference), it is tempting to speculate that the degree of EC proliferation dictates the vessel diameter. It is unclear, however, whether an increased EC proliferative index would correlate with the final vessel diameter *in vivo*.

Apart from the EC proliferative index, migratory properties and differentiation cues in the microenvironment contribute to the width of a developing vessel. Mice exclusively expressing the diffusible vascular endothelial growth factor-A120 isoform display enlarged vessels in the developing hindbrain with increased numbers of ECs/vessel length[11]. Furthermore, induced overexpression of activated Notch4 gives rise to wider vessels both in embryoid bodies and *in vivo*, even though EC proliferation is reduced[12]. Also, the Notch ligand jagged 1 is up-regulated in the presence of LM-111 during differentiation of bovine ECs, suggesting a link between the two molecular systems[13].

It is evident from the embryonic stem cell model, as well as from gene-targeted mouse models, that the vascular BM LMs play a role in establishment of the vessel architecture. However, LMs are not required for development of ECs per se[3]. Here we suggest that the balance of BM components plays an important role in regulation of EC proliferation and possibly other EC responses, which in turn dictates the vessel diameter. The vascular LMs may, therefore, in addition to serving as a scaffold and promoting cell attachment to avoid apoptosis, serve to control differentiation and restrict EC proliferation.

LM loss of function (LOF) studies have generated valuable *in vivo* data, but the knowledge is still incomplete. Detailed analysis of angiogenic tissues in the LM LOF mice may provide key information that is currently lacking. Furthermore, inducible LM LOFs would allow for analysis of the role of LMs in adult and pathological tissues. A key question is whether single BM components or fragments thereof play distinct roles in the angiogenic process, or if it is the balance between the different components of the BM that guides the morphology of the new vessel.

### ACKNOWLEDGMENTS

L.J. is supported by Cancer Research UK and a European Molecular Biology Organization postdoctoral fellowship. L.C.W. gratefully acknowledges funding from the Swedish Cancer Foundation and the Swedish Science Council.

### REFERENCES

1. Folkman, J. (1995) Angiogenesis in cancer, vascular, rheumatoid and other disease. *Nat. Med.* **1,** 27–31.
2. Baluk, P., Hashizume, H., and McDonald, D.M. (2005) Cellular abnormalities of blood vessels as targets in cancer. *Curr. Opin. Genet. Dev.* **15,** 102–111.
3. Jakobsson, L., Domogatskaya, A., Tryggvason, K., Edgar, D., and Claesson-Welsh, L. (2008) Laminin deposition is dispensable for vasculogenesis but regulates blood vessel diameter independent of flow. *FASEB J.* **22,** 1530–1539.
4. Sorokin, L.M., Pausch, F., Frieser, M., Kroger, S., Ohage, E., and Deutzmann, R. (1997) Developmental regulation of the laminin alpha5 chain suggests a role in epithelial and endothelial cell maturation. *Dev. Biol.* **189,** 285–300.
5. Miner, J.H. (2008) Laminins and their roles in mammals. *Microsc. Res. Tech.* **71,** 349–356.
6. Hallmann, R., Horn, N., Selg, M., Wendler, O., Pausch, F., and Sorokin, L.M. (2005) Expression and function of laminins in the embryonic and mature vasculature. *Physiol. Rev.* **85,** 979–1000.

7. Miner, J.H., Cunningham, J., and Sanes, J.R. (1998) Roles for laminin in embryogenesis: exencephaly, syndactyly, and placentopathy in mice lacking the laminin alpha5 chain. *J. Cell Biol.* **143,** 1713–1723.
8. Thyboll, J., Kortesmaa, J., Cao, R., Soininen, R., Wang, L., Iivanainen, A., Sorokin, L., Risling, M., Cao, Y., and Tryggvason, K. (2002) Deletion of the laminin alpha4 chain leads to impaired microvessel maturation. *Mol. Cell. Biol.* **22,** 1194–1202.
9. Jakobsson, L., Kreuger, J., and Claesson-Welsh, L. (2007) Building blood vessels--stem cell models in vascular biology. *J. Cell Biol.* **177,** 751–755.
10. Li, S., Harrison, D., Carbonetto, S., Fassler, R., Smyth, N., Edgar, D., and Yurchenco, P.D. (2002) Matrix assembly, regulation, and survival functions of laminin and its receptors in embryonic stem cell differentiation. *J. Cell Biol.* **157,** 1279–1290.
11. Ruhrberg, C., Gerhardt, H., Golding, M., Watson, R., Ioannidou, S., Fujisawa, H., Betsholtz, C., and Shima, D.T. (2002) Spatially restricted patterning cues provided by heparin-binding VEGF-A control blood vessel branching morphogenesis. *Genes Dev.* **16,** 2684–2698.
12. Uyttendaele, H., Ho, J., Rossant, J., and Kitajewski, J. (2001) Vascular patterning defects associated with expression of activated Notch4 in embryonic endothelium. *Proc. Natl. Acad. Sci. U. S. A.* **98,** 5643–5648.
13. Dixelius, J., Jakobsson, L., Genersch, E., Bohman, S., Ekblom, P., and Claesson-Welsh, L. (2004) Laminin-1 promotes angiogenesis in synergy with fibroblast growth factor by distinct regulation of the gene and protein expression profile in endothelial cells. *J. Biol. Chem.* **279,** 23766–23772.

---

**This article should be cited as follows:**

Jakobsson, L. and Claesson-Welsh, L. (2008) Vascular basement membrane components in angiogenesis — an act of balance. *TheScientificWorldJOURNAL* **8**, 1246–1249. DOI 10.1100/tsw.2008.155.

---
